# Multiphoton Microscopy for Ophthalmic Imaging

**DOI:** 10.1155/2011/870879

**Published:** 2011-01-03

**Authors:** Emily A. Gibson, Omid Masihzadeh, Tim C. Lei, David A. Ammar, Malik Y. Kahook

**Affiliations:** ^1^Department of Bioengineering, University of Colorado Denver, Denver, 12700 E. 19th Ave, Mail Stop 8607, Aurora, CO 80045, USA; ^2^Department of Electrical Engineering, University of Colorado Denver, Denver, CO 80217, USA; ^3^Department of Ophthalmology, School of Medicine, University of Colorado, 1675 Aurora Ct, Mail Stop F-731, Aurora, CO 80045, USA

## Abstract

We review multiphoton microscopy (MPM) including two-photon autofluorescence (2PAF), second harmonic generation (SHG), third harmonic generation (THG), fluorescence lifetime (FLIM), and coherent anti-Stokes Raman Scattering (CARS) with relevance to clinical applications in ophthalmology. The different imaging modalities are discussed highlighting the particular strength that each has for functional tissue imaging. MPM is compared with current clinical ophthalmological imaging techniques such as reflectance confocal microscopy, optical coherence tomography, and fluorescence imaging. In addition, we discuss the future prospects for MPM in disease detection and clinical monitoring of disease progression, understanding fundamental disease mechanisms, and real-time monitoring of drug delivery.

## 1. Introduction

Imaging modalities such as digital photography and ultrasound have become integral in the clinical and surgical practice of ophthalmology over the past few decades. More recently, diode laser-based imaging devices such as GDx (Carl Zeiss Meditec, Inc., Dublin, CA), Heidelberg Retinal Tomography (HRT, Heidelberg Engineering, Heidelberg, Germany), and optical coherence tomography (OCT) have become invaluable in the examination and early diagnosis of disease ranging from macular degeneration to glaucoma. Despite these advances, the aforementioned imaging devices are restricted in their ability to image tissue structure while being largely unable to elucidate tissue function. This limitation becomes even more important when noting that the structural normative databases used to delineate abnormal from normal tissue have inherent limitations. Physiologic differences from patient to patient as well as coexisting conditions, such as possible thinning of the retinal nerve fiber layer (RNFL) in high myopia, may alter the structure of tissues but often do not alter actual visual function [1]. An imaging modality that could combine both structural and functional imaging would allow physicians to make more informed decisions that directly relate to disruptions in visual performance. 

Multiphoton microscopy (MPM) has found increasing use in laboratory-based biomedical imaging due to its subcellular resolution along with the ability to obtain structural and functional information. These properties make MPM unique compared to other imaging modalities such as ultrasound, magnetic resonance imaging (MRI), or X-ray/computed tomography (CT) imaging. However, to achieve these benefits, there is a drawback in the limited tissue penetration depth as well as the ability to image highly scattering tissues such as sclera. Fortunately, there are opportunities for using optical imaging in the eye because of the transparency of the cornea, lens, and vitreous humor to visible and infrared light. In this paper, we review current research on MPM imaging of the eye and discuss future possibilities for applications to early disease diagnosis and monitoring of patient treatment outcomes.

## 2. Multiphoton Microscopy: Theory and Implementation

### 2.1. Overview of Multiphoton Microscopy

Multiphoton microscopy has been described in depth in many review articles and texts [2–7]. Here, we highlight some of the key features of MPM that would have application in clinical *in vivo* measurements. MPM is an imaging method based on nonlinear optical response of a medium, that is, processes that involve more than one photon interacting simultaneously with a molecule. Since the probability of simultaneous interactions with two (or more) photons is extremely low (cross-sections on order of 10^−50^ cm^4^ s or 1 GM), the process only occurs when there is high photon flux (on the order of 10^6^–10^8^ W/cm^2^) [2, 8]. This is typically achieved using a pulsed near-infrared laser with a pulse duration on order of *∼*100 femtoseconds focused with a high numerical aperture objective. As a result, MPM offers intrinsic axial cross-sectioning because the process only occurs at the focus of the microscope objective, where the laser intensity is greatest. MPM imaging offers equivalent resolution as confocal microscopy (*∼*200 nm lateral and *∼*1.0 micron axial) but does not require the use of a pinhole. Image acquisition times are similar to confocal microscopy, with an image of 256 × 256 pixels acquired at video rate. The acquisition time is comparable to OCT with a difference in that MPM provides subcellular resolution within a smaller-imaged region while OCT scans over a larger area with reduced resolution. It is important to realize, for applications in medicine, that MPM can provide contrast without exogenous dye labeling and is a completely noninvasive technique. 

An additional advantage of using a near-infrared laser source is deeper tissue penetration due to reduced light scattering with longer wavelengths of light. The imaging depth possible depends upon the type of tissue and the wavelength of the laser. Imaging depths of up to 1 mm have been reported in brain tissue by using higher laser powers from a Ti:sapphire regenerative amplifier at 800 nm [9]. More sophisticated methods for deeper image penetration employ adaptive wavefront correction, similar to the technology used by astronomers in ground-based telescopes. Analogous to the distortion of the image quality due to the atmosphere, in deep tissue MPM, the local inhomogeneities in refractive index in tissue distort the focus of the excitation laser, causing a reduction in signal. A deformable mirror or spatial light modulator can be incorporated before the objective to precompensate for the wavefront distortion leading to a diffraction-limited focal spot size in tissue depths of 500 *μ*m [10–12]. 

**Figure 1:**
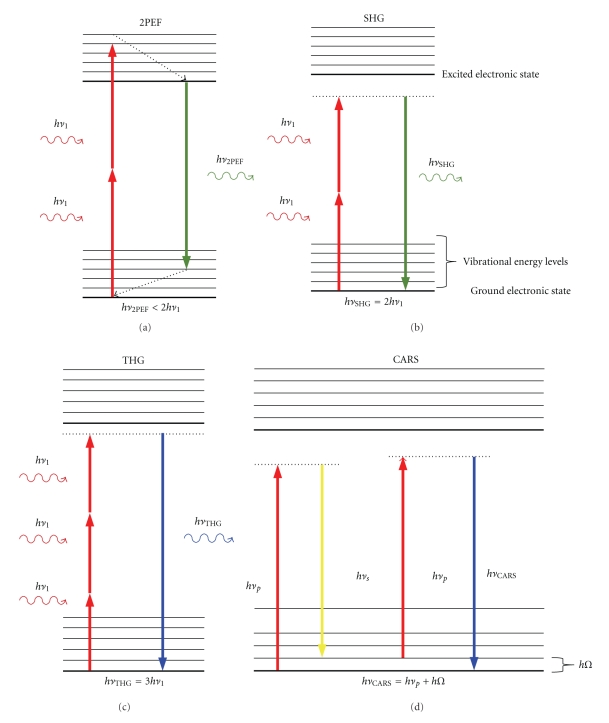
Jablonski diagram showing the interaction of multiple infrared photons with the electronic and vibrational energy levels of a molecule. (a) In two-photon excitation fluorescence (2PEF), the molecule absorbs two infrared photons that promote it to the excited electronic state. After relaxation to a lower vibrational level, the molecule emits a lower energy (red-shifted) photon. (b) In second harmonic generation (SHG), two infrared photons are instantaneously upconverted to a single photon of twice the energy. (c) In third harmonic generation (THG), three infrared photons are instantaneously upconverted to a single photon of thrice the energy. (d) In Coherent anti-Stokes Raman Scattering (CARS), two photons with energies *hν*_*p*_ and *hν*_*s*_ coherently excite the vibrational level with energy *hΩ* = *hν*_*p*_ − *hν*_*s*_. An additional photon, *hν*_*p*_, interacts with the vibrationally excited molecule emitting a photon with energy given by the original incident photon energy plus the vibrational energy, *hν*_CARS_ = *hν*_*p*_ + *hΩ*, leaving the molecule in its original ground state. (Note that photon energy is given by Planck's constant, *h*, multiplied by the frequency of the photon *ν*.)

MPM includes two-photon excitation fluorescence (2PEF), second harmonic generation (SHG), and third harmonic generation (THG), as well as coherent anti-Stokes Raman Scattering (CARS) described in a later section.  Figure 1 shows a schematic of the different processes that result from nonlinear multiphoton interactions with a molecule. 2PEF is very similar to traditional fluorescence, except that two photons of a lower energy are simultaneously absorbed to excite a fluorophore. When 2PEF is used to excite endogenous fluorophores such as elastin and NAD(P)H, it is typically called two-photon excitation autofluorescence (2PAF). A fluorophore is any molecule that can absorb photons and emit the energy as a photon with a red-shifted wavelength. Another nonlinear process that occurs with two-photon excitation is second harmonic generation (SHG). SHG can only occur when light interacts with noncentrosymmetric (asymmetric) macromolecular structures. Molecules such as collagen fibers can simultaneously “scatter” two lower-energy photons as a single photon of twice the energy. Third harmonic generation is analogous to SHG; however, in this case, three photons of the fundamental are upconverted to a single photon of three times the energy. THG only requires about ten times the photon flux as SHG and, therefore, can be a useful tool for imaging. THG highlights different features of a sample than SHG because it is generated at the interface of media with differing third-order nonlinear susceptibilities, *χ* ^(3)^ [13, 14].

Endogenous fluorophores have varying two-photon cross-sections as a function of wavelength and have been measured and reported [15, 16]. The center wavelength of a Ti:spphire laser can be tuned over a large spectral range from 700 to 1050 nm, making it an extremely useful source for two-photon autofluorescence excitation. In this manner, different compounds in tissue can be highlighted by tuning the excitation wavelength. For example, the two-photon cross-sections of many endogenous fluorophores peak below 700 nm and decrease at higher wavelengths, while SHG emission remains strong at longer wavelengths from 900 to 1000 nm [15]. By tuning the excitation laser to longer wavelengths, collagen structures in tissue can be distinguished from autofluorescence [17, 18]. In another example of the utility of excitation wavelength tuning, NAD(P)H was distinguished from FAD by dual wavelength excitation at 730 nm (where both compounds are excited) and at 900 nm (where FAD is exclusively excited, while NAD(P)H has a negligible two-photon cross-section) [16].  Table 1 gives a list of endogenous fluorophores and tissue structures and the imaging technique that provides the best contrast mechanism.

### 2.2. Optical Instrumentation

**Figure 2:**
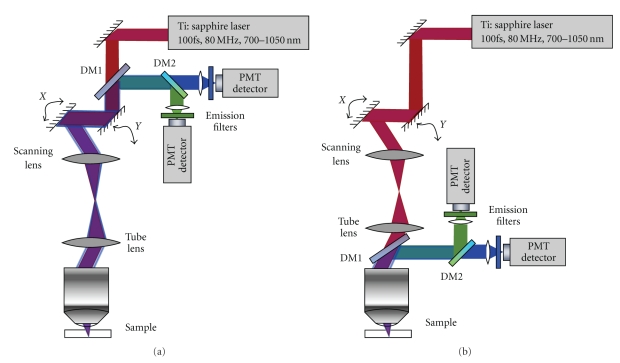
Schematic of elements of a multiphoton imaging optical setup. (a) Descanned detection whereby the emitted signal is collected after travelling back through the scanning mirrors. (b) Nondescanned detection. DM indicates dichroic mirror.

Both 2PAF and SHG can be simultaneously measured using the same optical setup since the SHG signal occurs at a distinct wavelength (exactly half the excitation wavelength) and can be separated from autofluorescence using spectral filtering.  Figure 2 shows a schematic of a typical setup for performing MPM. The apparatus consists of a pulsed femtosecond infrared laser source, typically Ti:sapphire mode-locked oscillator. The excitation light first passes through a two-axis galvo-scanning mirror stage and is imaged, using a scan lens and a tube lens, on to the back of the microscope objective. The microscope objective focuses the light to a focal volume typically around 200 nm axial and 1.0 microns lateral (depending upon the numerical aperture of the objective). The excitation volume is on order of a femtoliter (10^−15^ L). The generated two-photon signal is collected back through the same objective and separated from the excitation light using a dichroic mirror and filter. It is then imaged onto the front of a photomultiplier tube (PMT). In descanned detection, the multiphoton emission is relayed back through the galvo mirrors so that the scanning motion is cancelled out and the emitted light is stationary at the detector. In nondescanned detection, the emission light is separated using a dichroic mirror without passing through the scanning mirrors greatly reducing the loss in signal associated with reflections off of the mirrors and the lenses in the optical path. Because the two-photon emission is not passed back through the scanning mirrors, the emission light on the PMT moves during scanning; however, the PMT is typically insensitive to this motion because of its large detection area. Nondescanned detection is available only for multiphoton imaging because, unlike in single-photon confocal imaging, a pinhole is not required to eliminate out-of-focus light from the image.

### 2.3. Advanced Multiphoton Microscopy Techniques

**Figure 3:**
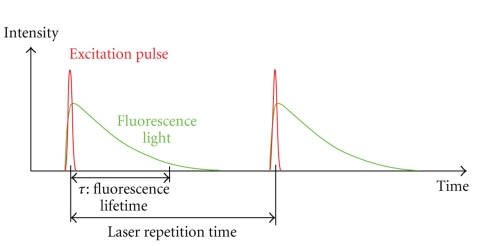
Illustration of the fluorescence lifetime principle. The short-pulsed excitation light (red) and the longer time duration fluorescence emission light (green) is shown as a function of time. In FLIM, the time scale of the fluorescence emission, *τ*, is measured.

**Figure 4:**
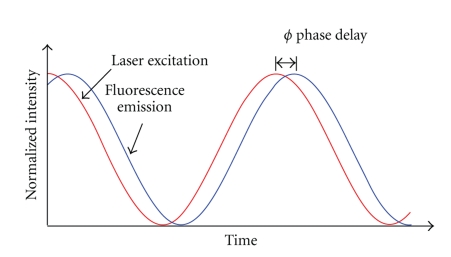
Illustration of frequency-domain fluorescence lifetime measurement. The excitation light (red) is modulated in amplitude at a frequency *ω*, while the fluorescence light (blue) is emitted with the same modulation frequency but with a phase shift in time, *ϕ.* For a single exponential lifetime, the value of the fluorescence lifetime is related by tan*ϕ* = *ωτ*.

Fluorescence lifetime imaging microscopy (FLIM) is an additional imaging technique that is better able to distinguish between the different endogenous fluorophores in a biological sample. Due to the broad and overlapping emission spectra of many endogenous fluorophores, it is difficult to quantitatively measure the concentrations of these different species contributing to the autofluorescence emission signal by spectral filtering alone. Fluorescence lifetime can also provide information on the surrounding environment of the fluorophore. FLIM is based on the fact that every fluorophore has a characteristic excited-state lifetime, *τ*, or time for the molecule to decay from the excited electronic state to the ground state. This decay is characterized by a single or multiple exponential (in the case of an inhomogeneous environment) of the form *P*(*t*) = *P*_0_∑_*i*=1_^*n*^ *A*_*i*_exp (−*t*/*τ*_*i*_), where *P*(*t*) is the population in the excited state as a function of time. Here, *P*_0_ is the initial population in the excited state, and *A*_*i*_ is the normalized amplitude of the exponential component with lifetime *τ*_*i*_. Fluorescence lifetime signal from a biological sample containing multiple fluorophores can become further complicated. For multiple exponential lifetimes, the average lifetime value is sometimes reported, given by $\overset{®}{\tau }=\sum_{i=1}^{n}A_{i}\tau_{i}$. This lifetime information can be measured either by time-domain or frequency-domain methods [20–22]. In the time-domain technique, a pulsed excitation source is used to excite the fluorophore of interest in the biological sample. The subsequent time profile of the fluorescence emission is measured using time gating techniques. Figure 3 illustrates the time-domain FLIM process. In frequency-domain FLIM, an amplitude-modulated excitation source is employed. The lifetime of the fluorophore causes the emitted fluorescence signal to be modulated at the same frequency but with a phase shift relative to the excitation light (see Figure 4). Measurement of this phase offset using phase-sensitive detection (such as a lockin amplifier) will then give the value of the lifetime, *τ*, by the relation tan*ϕ* = *ωτ*, where *ϕ* is the phase offset, *ω* is the modulation frequency. If the lifetime is multiexponential, it is necessary to measure the phase offset at several modulation frequencies in order to obtain the different lifetime components [23]. Some advantages of the frequency-domain technique include faster acquisition compared to the time-domain technique and insensitivity to high photon count rates, which is a problem with time-domain techniques as high count rates can skew the time histogram to shorter times. Frequency-domain FLIM has been recently demonstrated using an inexpensive field-programmable gate array and photon-counting detection giving very rapid and highly sensitive measurements [24].

FLIM has found particular use in imaging NADH/NAD(P)H. Bound and unbound NADH have different characteristic fluorescence lifetimes (free NADH ~0.3 ns, protein-bound NADH ~2 ns), and therefore FLIM can be used to measure the ratios of these populations giving an indication of metabolic activity and oxidative stress [25–27]. For example, several studies have demonstrated differences in NADH lifetime values between cancerous and normal cells. FLIM is being actively researched for clinical screening of the skin for distinguishing between dysplastic nevi, melanoma, and other dermatological disorders [28–30]. Recently, FLIM was employed to study the cornea using autofluorescence lifetime imaging [31]. In ophthalmological applications, FLIM can be potentially utilized in studying oxidative stress in particular due to interaction of tissue with pharmacological agents or due to disease.

An additional multiphoton imaging technique that is fundamentally different from both fluorescence and harmonic generation is coherent anti-Stokes Raman Spectroscopy (CARS). CARS is a nonlinear version of Raman spectroscopy. In the Raman process, a narrow-band laser illuminates the sample, and a portion of the incident photons are scattered by interactions with molecular vibrations, resulting in a shift to higher (anti-Stokes) or lower frequency (Stokes) photons. The signal intensity is very weak because of the extremely low-scattering cross-section (*∼*10^−30^ cm^2^/molecule) as opposed to the absorption cross-section of a typical fluorophore (*∼*10^−15^ cm^2^/molecule). 

In contrast to traditional Raman spectroscopy, CARS is a nonlinear optical process that selectively and coherently excites vibrational resonances of biomolecules to rapidly obtain the Raman (vibrational) spectrum. Compared to traditional Raman scattering, the CARS process increases the detection sensitivity by up to 10^7^ to allow rapid data acquisition [32]. With the associated decrease in measurement times, CARS has been applied in biomedical microscopy to image live cells at video rates without extrinsic fluorescence dye labeling [32–34]. Figure 1 illustrates the CARS process. Two photons (pump and Stokes) excite a specific vibrational resonance coherently. A third photon (probe) subsequently measures the density of the vibrational resonance. The number of emitted anti-Stokes photons that are energy shifted by that vibrational mode is proportional to the square of the density of the vibrational oscillators, thus yielding the molecular concentration of the target [35]. A traditional CARS setup uses two synchronized picosecond lasers or a single picosecond laser with an optical parametric amplifier to generate the two laser beams with different frequencies matched to one particular vibrational resonance. By tuning the laser frequency difference to a particular vibrational mode, for example, 2850 cm^−1^ of the CH_2_ stretch for lipids, chemical-specific imaging can be achieved all without use of endogenous dyes or other labeling techniques. CARS therefore has great potential clinical applications, although it has not yet been applied to imaging in the eye.

### 2.4. Microendoscopy

Due to the limitations in the penetration depth, MPM has so far only been applied in the clinic for screening of the skin [28, 37]. A major step towards extending the applicability of MP imaging for clinically relevant applications is the introduction of microendoscopy for intrabody tissue imaging inaccessible to standard bulky optics, [38–43]. Development of microendoscopes or flexible probes for MP imaging can greatly improve the instrumentation for clinical use in ophthalmology. Several demonstrated research applications of microendoscopy include probing of neural activity [39, 40, 44], blood flow measurements [38, 45], and imaging of goblet cells in gastric epithelium [46]. Furthermore, clinical high-resolution microendoscopy has been demonstrated to detect the extracellular matrix proteins collagen and elastin in the human dermis [47, 48]. Imaging of the eye in animal models using a microendoscope has been demonstrated by Wang et al. [18].

The introduction of compound gradient refractive index (GRIN) lenses as focusing optics [44, 49, 50], double-clad photonic crystal fibers [51, 52] for superior detection efficiency and mechanical flexibility, and microelectromechanical systems (MEMS) scanning mirrors [52–54] has been among the most important technological advancements towards microendoscopy. The majority of micro-lenses used in nonlinear imaging, GRIN lenses, have a typical size of 0.2–1 mm in diameter, 1–10 cm in length, and a numerical aperture of less than 0.6. However, due to low numerical aperture and optical aberration, the optical Rayleigh resolution has been limited to *∼*1 *μ*m in lateral and *∼*10 *μ*m in axial direction [39, 40]. Recently, aberration-corrected, high-NA planoconvex lenses (NA < 0.85) acting like micro-objectives have been reported to provide on-axis resolution comparable to water-immersion objectives [50]. With further advances, microendoscopy technology can bring the full capabilities of MPM to clinical imaging.

### 2.5. Histology

Another clinical application of MPM is in histology, where there is no requirement for deep tissue penetration as the tissue can easily be sectioned in 10–100 *μ*m thick slices. MPM can have advantages over traditional histological staining techniques by providing more detailed information and highlighting features without perturbing the sample through processing. Preparation of samples for both standard histological staining and electron microscopy require chemical fixation and dehydration with alcohols. These treatments can cause artifacts and distortions within the tissue due to infusion of fixatives and shrinkage of tissue due to alcohol treatment. In addition, changes to fine tissue morphology can occur with heat infusion of paraffin (for histology) or with polymerization of resin (EM). The application of MPM in histology can be immediately implemented in the clinic and is greatly underutilized. There are opportunities for the development of more accessible MPM systems that would perform imaging on tissue samples with automated analysis routines to aid physicians.

## 3. Multiphoton Imaging of the Eye

### 3.1. Comparison of Multiphoton to Current Clinical Imaging Modalities

Several groups have preformed MP imaging of different regions of the eye, *ex vivo*, implicated in a variety of disease pathologies [31, 55–68]. These studies show that MP imaging of the eye for clinical applications has great promise. Current clinical techniques for imaging include optical coherence tomography (OCT) and confocal reflectance microscopy as well as fluorescence imaging. In comparison with MP imaging, OCT imaging has poorer spatial resolution of 2–10 *μ*m lateral and therefore cannot be used to reveal subcellular level structure. While confocal reflectance microscopy does allow subcellular level resolution, its contrast mechanism is due to changes in index of refraction, and therefore it does not have the functional information inherent in MP imaging. Fluorescence imaging uses exogenous dyes to stain the eye in a nonspecific manner typically for looking at the vasculature in the retina. None of these devices are capable of providing functional data for imaged tissues and are thus limited in their ability to direct or influence clinical decision making on a consistent basis.

### 3.2. Multiphoton Imaging of Eye Sections

**Figure 5:**
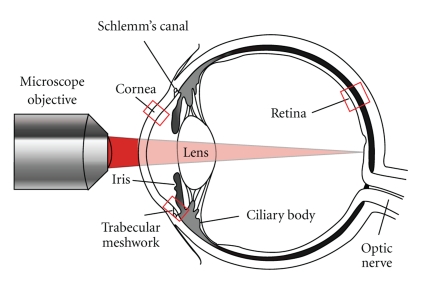
Schematic of the eye highlighting the regions of interest for imaging with multiphoton microscopy. Light path for imaging of the retina through the anterior chamber and lens is shown.

Figure 5 shows a diagram of the eye highlighting the regions of interest for MPM studies. MP imaging of the cornea is of interest for diagnosis of diseases such as corneal dystrophies and endothelial dysfunction and has been reported by several groups. Steven et al. demonstrated 2PAF, SHG, and autofluorescence lifetime imaging of different ocular surface pathologies on corneal tissue sections using a commercial instrument for clinical MP imaging (DermaInspect, JenLab GmbH, Neuengönna, Germany) [31]. By performing multiple wavelength excitation at 730 nm and 835 nm and resolving different lifetime components by FLIM, they were able to identify and distinguish between epithelial cells, goblet cells, erythrocytes, macrophages, collagen, elastin, vascular structures, and pigmented lesions. Aptel et al. demonstrated SHG, 2PAF, and THG of corneal tissue sections and SHG and 2PAF of the trabecular meshwork. In particular, they demonstrated an additional contrast mechanism by selecting either linear or circularly polarized excitation for THG [55]. Chen et al. demonstrated simultaneous reflectance confocal microscopy, 2PAF, and SHG on corneal sections [56, 57]. Morishige et al. used three-dimensional SHG imaging to characterize structural lamellar organization of the anterior cornea [58]. Teng et al. demonstrated simultaneous SHG and 2PAF imaging in an intact *ex vivo* porcine eye to identify cellular components of the cornea, limbus, and conjunctiva, as well as imaging corneal and scleral collagen fibers [59]. MP imaging of both cornea and retinal sections was demonstrated by Wang et al. [60, 61].

MP imaging of the retina has also been demonstrated and may find utility in detection of retinal pigment epithelium (RPE) dysfunction and photoreceptor-related dystrophies. To date, no imaging of the human retina has been performed through the anterior chamber, although explants of human retina and RPE have been imaged by the tissue autofluorescence [62–64]. There are additional difficulties in imaging the retina for clinical applications due to the optical constraints posed by the iris that effectively limit the numerical aperture. For example, for an iris opening of 8 mm diameter and typical distance from iris to the retina of 17 mm, the effective numerical aperture, which is indicative of the collection angle of the emitted optical signal, is given by the equation NA = *n*sin*θ* ~ 0.3, using the index of refraction of water (*n* = 1.33). The numerical aperture besides limiting the collection efficiency of the emitted signal also limits how tightly the excitation light can be focused, thus determining the achievable resolution of the image. In addition, the aberrations in the lens of the eye can also decrease the obtainable resolution in MP imaging. In order to alleviate this problem, wavefront correction using adaptive optics has been performed for retinal imaging [65, 66].

**Figure 6:**
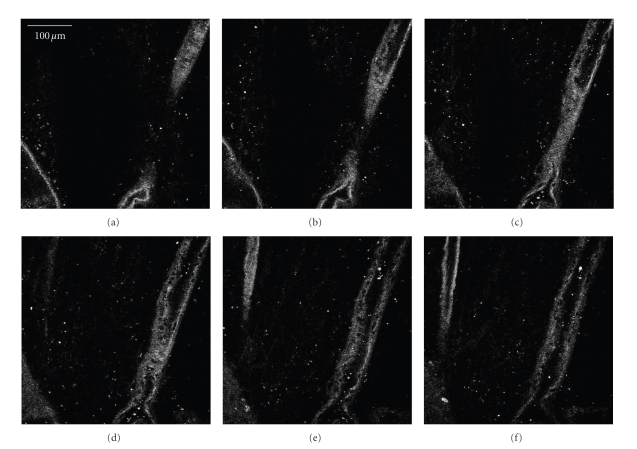
Vascular bed of a human retina imaged by second harmonic generation (SHG). Serial *z*-sections, spaced 12 *μ*m apart, of a human retina are shown beginning with (a) through (f). The images shown are collected using the 800 nm near infrared laser excitation with a collection window of 390–410 nm. The collagen structure of a large blood vessel is clearly visible through the series, which represents a height of 60 *μ*m.

To our knowledge, MP imaging of a living retina/RPE has only been performed in a rodent eye by imaging through the exterior sclera. In this instance, Imanishi et al. used MPM to view the retina/RPE autofluorescence as well as to localize stores of the visual pigment retinal [67]. The retina itself has no apparent SHG signal, although the overlying retinal vasculature and underlying connective tissue can be imaged via the collagen content. We have demonstrated this in our lab on a Zeiss LSM510 multiphoton confocal microscope, illustrated in Figure 6. In Figure 6(c), one can see the top of the blood vessel followed by the inside of the vessel as the objective moves through the vascular bed. Further optical sectioning past this point yields no further SHG signal.

**Figure 7:**
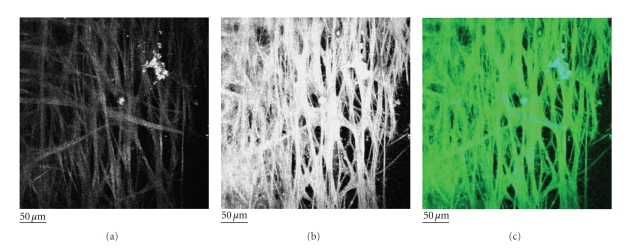
Second harmonic generation (SHG) and two-photon autofluorescence (2PAF) of TM region of a human eye from a 73-year-old donor. A section of the eye was flat-mounted with the anterior chamber facing the microscope objective. Images represent a projection of the multiple *z*-sections flattened into a single plane. (a) The SHG emission (388 nm to 409 nm) collected from 800 nm excitation of TM. (b) The 2PAF emission window (452 nm to 644 nm) collected simultaneously. (c) Merged image of SHG (blue) and AF (green) emission. Black scale bar = 50 *μ*m. This figure is reprinted from [36].

Recently, we have demonstrated MP imaging of the trabecular meshwork (TM) region of the eye using SHG and 2PAF [68]. Imaging of the TM is important because degeneration of the TM is implicated in glaucoma; therefore, characterizing the cell and collagen structures in the TM may allow early diagnosis, disease monitoring, as well as fundamental studies of the disease mechanism. In our paper, the TM was flat-mounted and visually sectioned by 0.5 *μ*m intervals to a depth of 50 *μ*m and then computer modeled into a single-plane projection (Figure 7). SHG and 2PAF emission windows were collected using the META spectral detector on a Zeiss LSM510 multiphoton confocal system. Figures 7(a) and 7(b) show the SHG and 2PAF fluorescence, respectively. Although the SHG signal is comparatively weaker than the 2PAF, these two signals are qualitatively the same when overlapped in Figure 7(c) (blue = SHG, green = 2PAF). Since collagen is the most common noncentrosymmetric macromolecule in the TM, the SHG signal is highly suggestive of the fact that the structures seen by 2PAF are in fact collagen fibers. In these images, the majority of collagen fibers of the TM appear as smooth bundles of between 10 and 20 *μ*m, although the occasional *∼*1 *μ*m collagen fiber is visible. These bundles have a fairly consistent diameter over short distances but over longer distances (>250 *μ*m) commonly split or join other bundles. The end result is a meshwork of collagen interwoven with varying-sized regions of nonfluorescent signal, which we assume to be fluid spaces.

### 3.3. Transscleral Imaging

Issues for MPM use in the clinic include accessibility of the different regions of the eye to optical light. Imaging of the trabecular meshwork using MPM would be of great clinical value in particular for early disease diagnosis and monitoring of glaucoma. However, in order to access this region, it would be necessary to image through approximately 600 microns of scleral tissue. For transscleral imaging, in general, only the surface of the sclera can be imaged as the highly scattering scleral tissue greatly limits optical light transmission. Vogel et al. measured the optical properties of human sclera using an integrating sphere. They found a transmission of 6% at 442 nm, 35% at 804 nm, and 53% at 1064 nm [69]. Although the excitation light for MPM ranging from 800 to 1000 nm can likely penetrate the sclera, the shorter wavelength SHG and autofluorescence emission will be greatly reduced upon collection in the epidirection. We continue to research this known limitation of MPM for TM imaging and believe that future advances will lead to greater clinical applicability of this technology.

### 3.4. Power and Wavelength Requirements for In Vivo Imaging

One of the advantages of MPM is the ability to use infrared light illumination, which is much less phototoxic for the eye than visible light. One issue with this technique for clinical use is potential thermal mechanical damage to the tissue during imaging due to absorption of the laser energy and local heating effects. Several studies have shown that the thermal damage due to two-photon absorption in most tissue under imaging conditions is negligible [70]. However, there are potential damage issues associated with pigmented tissue such as those found in the skin or the retina. There are methods to mitigate this including reducing the repetition rate of the laser to allow for thermal diffusion between pulses on the same location in the tissue [71, 72]. The pulse duration of the laser can also have a great impact on thermal damage. Du et al. found a reduction in tissue damage threshold with pulse duration for the same total energy delivered to the tissue sample [73]. The differences in laser system parameters combined with differences in tissue type suggest that, before any clinical use of MPM, photodamage issues must be carefully characterized.

### 3.5. Drug Delivery Monitoring

Finally, one exciting future application of MPM is in monitoring drug delivery *in vivo*. Kek et al. applied two-photon microscopy to monitor the transscleral delivery of tazarotenic acid using its intrinsic fluorescence at 500 nm [74]. The emerging technique of stimulated Raman scattering (SRS) a similar multiphoton technique to CARS imaging also has great potential for drug delivery monitoring because of its specificity; that is, the generated signal is specific to a single chemical compound, as well as the linear dependence of the signal on concentration [75]. SRS has currently been applied to monitor penetration of dimethyl sulfoxide (DMSO), a skin-penetration enhancer and retinoic acid in the upper dermal layer. There are many opportunities for applying SRS to monitoring drug delivery in the eye due to the transparency of the tissue making deeper penetration depths possible as compared to the skin.

## 4. Conclusion and Future Prospects

Current imaging techniques, such as ultrasound and OCT, have greatly influenced the standards of clinical and surgical ophthalmic care. Physicians can now detect disease using very sensitive imaging modalities and can also follow the progression of disease, thus shedding light on the efficacy of applied interventions. While availability of fine structural information is increasingly available in the clinical setting, the actual function of the imaged structures remains unknown. MPM offers the potential for obtaining both structural and functional data on a wide range of ophthalmic tissues. For example, it may be possible to image the trabecular meshwork structure while also establishing the metabolism of individual trabecular meshwork cells by quantifying NAD(P)H concentrations in real time. Such information could lead to earlier and more precise disease detection, while also allowing for more insight into the effects of therapeutic interventions aimed at preserving vision. 

Future applicability of MPM in practice will require further advances in the ability to penetrate past tissues, such as sclera, that have high scattering properties. The safety of using MPM also requires further studies since some ocular tissues have high melanin content which may lead to greater energy absorption and related tissue damage. Another obstacle that will need to be addressed is the difficulty in obtaining data across the relatively long axial length distance noted between the surface of the cornea and the posterior pole. Fortunately, advances in MPM continue to develop at a rapid pace, and obstacles that existed in the past have been overcome with continued research. With continued advances, the application of MPM in ophthalmic practice promises to yield valuable clinical information that will ultimately result in improved patient care, which is the common goal of researchers and physicians alike.

## Figures and Tables

**Table 1:** Optimal imaging contrast mechanism for different biological molecules.

Compound	Imaging technique (excitation/emission wavelengths)
NAD(P)H	2PAF/FLIM (excitation 700–730 nm/emission peak 460 nm) [16]

FAD	2PAF/FLIM (excitation 700–900 nm/emission peak 525 nm) [16]

Elastin	2PAF (excitation 700–740 nm/emission peak 400 nm) [15, 19]

Collagen	SHG (SHG excitation is tunable/emission at one-half the excitation wavelength) [15]

Lipids	THG/CARS (THG excitation is tunable with emission at one-third the excitation wavelength) [14]

## References

[1] Kang SH, Hong SW, Im SK, Lee SH, Ahn MD (2010). Effect of myopia on the thickness of the retinal nerve fiber layer measured by Cirrus HD optical coherence tomography. *Investigative Ophthalmology & Visual Science*.

[2] So PTC, Dong CY, Masters BR, Berland KM (2000). Two-photon excitation fluorescence microscopy. *Annual Review of Biomedical Engineering*.

[3] König K (2000). Multiphoton microscopy in life sciences. *Journal of Microscopy*.

[4] Helmchen F, Denk W (2005). Deep tissue two-photon microscopy. *Nature Methods*.

[5] Squier J, Müller M (2001). High resolution nonlinear microscopy: a review of sources and methods for achieving optimal imaging. *Review of Scientific Instruments*.

[6] Zipfel WR, Williams RM, Webb WW (2003). Nonlinear magic: multiphoton microscopy in the biosciences. *Nature Biotechnology*.

[7] Masters BR, So PTC (2008). *Handbook of Biomedical Nonlinear Optical Microscopy*.

[8] Denk W, Strickler JH, Webb WW (1990). Two-photon laser scanning fluorescence microscopy. *Science*.

[9] Theer P, Hasan MT, Denk W (2003). Two-photon imaging to a depth of 1000 *μ*m in living brains by use of a Ti:Al2O3 regenerative amplifier. *Optics Letters*.

[10] Marsh PN, Burns D, Girkin JM (2003). Practical implementation of adaptive optics in multiphoton microscopy. *Optics Express*.

[11] Neil MAA, Juškaitis R, Booth MJ, Wilson T, Tanaka T, Kawata S (2000). Adaptive aberration correction in a two-photon microscope. *Journal of Microscopy*.

[12] Rueckel M, Mack-Bucher JA, Denk W (2006). Adaptive wavefront correction in two-photon microscopy using coherence-gated wavefront sensing. *Proceedings of the National Academy of Sciences of the United States of America*.

[13] Müller M, Squier J, Wilson KR, Brakenhoff GJ (1998). 3D microscopy of transparent objects using third-harmonic generation. *Journal of Microscopy*.

[14] Débarre D, Supatto W, Pena A-M (2006). Imaging lipid bodies in cells and tissues using third-harmonic generation microscopy. *Nature Methods*.

[15] Zipfel WR, Williams RM, Christiet R, Nikitin AY, Hyman BT, Webb WW (2003). Live tissue intrinsic emission microscopy using multiphoton-excited native fluorescence and second harmonic generation. *Proceedings of the National Academy of Sciences of the United States of America*.

[16] Huang S, Heikal AA, Webb WW (2002). Two-photon fluorescence spectroscopy and microscopy of NAD(P)H and flavoprotein. *Biophysical Journal*.

[17] König K, Schenke-Layland K, Riemann I, Stock UA (2005). Multiphoton autofluorescence imaging of intratissue elastic fibers. *Biomaterials*.

[18] Wang B-G, König K, Halbhuber K-J (2010). Two-photon microscopy of deep intravital tissues and its merits in clinical research. *Journal of Microscopy*.

[19] Alfano RR, Katz A Non invasive fluorescence-based instrumentation for cancer and precancer detection and screening.

[20] French T, So PTC, Dong CY, Berland KM, Gratton E (1998). Fluorescence lifetime imaging techniques for microscopy. *Methods in Cell Biology*.

[21] Gratton E, Breusegem S, Sutin J, Ruan Q, Barry N (2003). Fluorescence lifetime imaging for the two-photon microscope: time-domain and frequency-domain methods. *Journal of Biomedical Optics*.

[22] Lakowicz J (2006). *Principles of Fluorescence Spectroscopy*.

[23] Lakowicz JR (2006). Frequency-domain lifetime measurements. *Principles of Fluorescence Spectroscopy*.

[24] Colyer RA, Lee C, Gratton E (2008). A novel fluorescence lifetime imaging system that optimizes photon efficiency. *Microscopy Research and Technique*.

[25] Yu Q, Heikal AA (2009). Two-photon autofluorescence dynamics imaging reveals sensitivity of intracellular NADH concentration and conformation to cell physiology at the single-cell level. *Journal of Photochemistry and Photobiology B: Biology*.

[26] Lakowicz JR, Szmacinski H, Nowaczyk K, Johnson ML (1992). Fluorescence lifetime imaging of free and protein-bound NADH. *Proceedings of the National Academy of Sciences of the United States of America*.

[27] Bird DK, Yan L, Vrotsos KM (2005). Metabolic mapping of MCF10A human breast cells via multiphoton fluorescence lifetime imaging of the coenzyme NADH. *Cancer Research*.

[28] König K (2008). Clinical multiphoton tomography. *Journal of biophotonics*.

[29] De Beule PAA, Dunsby C, Galletly NP (2007). A hyperspectral fluorescence lifetime probe for skin cancer diagnosis. *Review of Scientific Instruments*.

[30] König K, Riemann I (2003). High-resolution multiphoton tomography of human skin with subcellular spatial resolution and picosecond time resolution. *Journal of Biomedical Optics*.

[31] Steven P, Müller M, Koop N, Rose C, Hüttmann G (2009). Comparison of Cornea Module and DermaInspect for noninvasive imaging of ocular surface pathologies. *Journal of biomedical optics*.

[32] Evans CL, Xie XS (2008). Coherent anti-Stokes Raman scattering microscopy: chemical imaging for biology and medicine. *Annual Review of Analytical Chemistry*.

[33] Evans CL, Potma EO, Puoris’haag M, Côté D, Lin CP, Xie XS (2005). Chemical imaging of tissue in vivo with video-rate coherent anti-Strokes Raman scattering microscopy. *Proceedings of the National Academy of Sciences of the United States of America*.

[34] Zumbusch A, Holtom GR, Xie XS (1999). Three-dimensional vibrational imaging by coherent anti-Stokes Raman scattering. *Physical Review Letters*.

[35] Cheng J-X, Volkmer A, Book LD, Xie XS (2001). Epi-detected coherent anti-stokes Raman scattering (E-CARS) microscope with high spectral resolution and high sensitivity. *Journal of Physical Chemistry B*.

[36] Ammar DA, Lei TC, Gibson EA, Kahook MY (2010). Two-photon imaging of the trabecular meshwork. *Molecular Vision*.

[37] König K, Speicher M, Bückle R (2009). Clinical optical coherence tomography combined with multiphoton tomography of patients with skin diseases. *Journal of Biophotonics*.

[38] Helmchen F, Fee MS, Tank DW, Denk W (2001). A miniature head-mounted two-photon microscope: high-resolution brain imaging in freely moving animals. *Neuron*.

[39] Jung JC, Mehta AD, Aksay E, Stepnoski R, Schnitzer MJ (2004). In vivo mammalian brain imaging using one- and two-photon fluorescence microendoscopy. *Journal of Neurophysiology*.

[40] Levene MJ, Dombeck DA, Kasischke KA, Molloy RP, Webb WW (2004). In vivo multiphoton microscopy of deep brain tissue. *Journal of Neurophysiology*.

[41] Bao H, Boussioutas A, Jeremy R, Russell S, Gu M (2010). Second harmonic generation imaging via nonlinear endomicroscopy. *Optics Express*.

[42] Bird D, Gu M (2003). Two-photon fluorescence endoscopy with a micro-optic scanning head. *Optics Letters*.

[43] Légaré F, Evans CL, Ganikhanov F, Xie XS (2006). Towards CARS endoscopy. *Optics Express*.

[44] Jung JC, Schnitzer MJ (2003). Multiphoton endoscopy. *Optics Letters*.

[45] Flusberg BA, Jung JC, Cocker ED, Anderson EP, Schnitzer MJ (2005). In vivo brain imaging using a portable 3.9 gram two-photon fluorescence microendoscope. *Optics Letters*.

[46] Bao H, Boussioutas A, Reynolds J, Russell S, Gu M (2009). Imaging of goblet cells as a marker for intestinal metaplasia of the stomach by one-photon and two-photon fluorescence endomicroscopy. *Journal of biomedical optics*.

[47] König K, Weinigel M, Hoppert D (2008). Multiphoton tissue imaging using high-NA microendoscopes and flexible scan heads for clinical studies and small animal research. *Journal of biophotonics*.

[48] König K, Ehlers A, Riemann I, Schenkl S, Bückle R, Kaatz M (2007). Clinical two-photon microendoscopy. *Microscopy Research and Technique*.

[49] Göbel W, Kerr JND, Nimmerjahn A, Helmchen F (2004). Miniaturized two-photon microscope based on a flexible coherent fiber bundle and a gradient-index lens objective. *Optics Letters*.

[50] Barretto RPJ, Messerschmidt B, Schnitzer MJ (2009). In vivo fluorescence imaging with high-resolution microlenses. *Nature Methods*.

[51] Fu L, Gan X, Gu M (2005). Nonlinear optical microscopy based on double-clad photonic crystal fibers. *Optics Express*.

[52] Fu L, Jain A, Xie H, Cranfield C, Gu M (2006). Nonlinear optical endoscopy based on a double-clad photonic crystal fiber and a MEMS mirror. *Optics Express*.

[53] Fu L, Jain A, Cranfield C, Xie H, Gu M (2007). Three-dimensional nonlinear optical endoscopy. *Journal of Biomedical Optics*.

[54] Piyawattanametha W, Barretto RPJ, Ko TH (2006). Fast-scanning two-photon fluorescence imaging based on a microelectromechanical systems two-dimensional scanning mirror. *Optics Letters*.

[55] Aptel F, Olivier N, Deniset-Besseau A (2010). Multimodal nonlinear imaging of the human cornea. *Investigative Ophthalmology & Visual Science*.

[56] Chen WL, Chou CK, Lin MG (2009). Single-wavelength reflected confocal and multiphoton microscopy for tissue imaging. *Journal of Biomedical Optics*.

[57] Chen W-L, Sun Y, Lo W, Tan H-Y, Dong C-Y (2008). Combination of multiphoton and reflective confocal imaging of cornea. *Microscopy Research and Technique*.

[58] Morishige N, Wahlert AJ, Kenney MC (2007). Second-harmonic imaging microscopy of normal human and keratoconus cornea. *Investigative Ophthalmology and Visual Science*.

[59] Teng S-W, Tan H-Y, Peng J-L (2006). Multiphoton autofluorescence and second-harmonic generation imaging of the ex vivo porcine eye. *Investigative Ophthalmology and Visual Science*.

[60] Wang B-G, Eitner A, Lindenau J, Halbhuber K-J (2008). High-resolution two-photon excitation microscopy of ocular tissues in porcine eye. *Lasers in Surgery and Medicine*.

[61] Wang B-G, Koenig K, Riemann I, Krieg R, Halbhuber K-J (2006). Intraocular multiphoton microscopy with subcellular spatial resolution by infrared femtosecond lasers. *Histochemistry and Cell Biology*.

[62] Bindewald-Wittich A, Han M, Schmitz-Valckenberg S (2006). Two-photon-excited fluorescence imaging of human RPE cells with a femtosecond Ti:sapphire laser. *Investigative Ophthalmology and Visual Science*.

[63] Han M, Giese G, Schmitz-Valckenberg S (2007). Age-related structural abnormalities in the human retina-choroid complex revealed by two-photon excited autofluorescence imaging. *Journal of Biomedical Optics*.

[64] Han M, Bindewald-Wittich A, Holz FG (2006). Two-photon excited autofluorescence imaging of human retinal pigment epithelial cells. *Journal of Biomedical Optics*.

[65] Gualda EJ, Bueno JM, Artal P (2010). Wavefront optimized nonlinear microscopy of ex vivo human retinas. *Journal of biomedical optics*.

[66] Burns SA, Tumbar R, Elsner AE, Ferguson D, Hammer DX (2007). Large-field-of-view, modular, stabilized adaptive-optics-based scanning laser ophthalmoscope. *Journal of the Optical Society of America A: Optics and Image Science, and Vision*.

[67] Imanishi Y, Batten ML, Piston DW, Baehr W, Palczewski K (2004). Noninvasive two-photon imaging reveals retinyl ester storage structures in the eye. *Journal of Cell Biology*.

[68] Ammar DA, Lei TC, Gibson EA, Kahook MY (2010). Two-photon imaging of the trabecular meshwork. *Molecular Vision*.

[69] Vogel A, Dlugos C, Nuffer R, Birngruber R (1991). Optical properties of human sclera, and their consequences for transscleral laser applications. *Lasers in Surgery and Medicine*.

[70] Hopt A, Neher E (2001). Highly nonlinear photodamage in two-photon fluorescence microscopy. *Biophysical Journal*.

[71] Masters BR, So PTC, Buehler C (2004). Mitigating thermal mechanical damage potential during two-photon dermal imaging. *Journal of Biomedical Optics*.

[72] Denk W, Piston D, Webb WW, Pawley JB (2006). Two-photon molecular excitation laser-scanning microscopy. *Handbook of Biological Confocal Microscopy*.

[73] Du D (1995). *Damage Threshold as a Function of Pulse Duration in Biological Tissue*.

[74] Kek WK wo-photon fluorescence excitation microscopy to assess trans-scleral diffusional pathways in an isolated perfused bovine eye model.

[75] Freudiger CW, Min W, Saar BG (2008). Label-free biomedical imaging with high sensitivity by stimulated raman scattering microscopy. *Science*.

